# Enabling surface dependent diffusion in spatial simulations using Smoldyn

**DOI:** 10.1186/s13104-015-1723-6

**Published:** 2015-12-08

**Authors:** Christine Seeliger, Nicolas Le Novère

**Affiliations:** European Bioinformatics Institute, Wellcome Trust Genome Campus, Cambridge, CB10 1SD UK; The Babraham Institute, Babraham Research Campus, Babraham, CB22 3AT UK

**Keywords:** Simulation, Stochastic, Single Particle, Spatial, Diffusion, Smoldyn, Membrane, Surface

## Abstract

**Background:**

Spatial computer simulations are becoming more feasible and relevant for studies of signaling pathways due to technical advances in experimental techniques yielding better high resolution data. However, many common single particle simulation environments used in computational systems biology lack the functionality to easily implement spatially heterogeneous membrane environments.

**Results:**

We introduce an extension to the single particle simulator Smoldyn that allows modeling of surface-dependent diffusion, without unnecessarily increasing molecular states or numbers, hence avoiding explosion of molecule and reaction definitions.

**Conclusions:**

We demonstrate the usefulness of this approach studying AMPA receptor diffusion at the postsynaptic density and its spatial trapping without introducing hypothetical scaffold elements or membrane barriers.

**Electronic supplementary material:**

The online version of this article (doi:10.1186/s13104-015-1723-6) contains supplementary material, which is available to authorized users.

## Findings

Computational modeling is increasingly used to study the dynamic behavior of complex signaling systems in time and space. Different modeling approaches, and with them simulation environments, exist to allow the development, implementation and analysis of signaling models. With improvements in experimental techniques, detailed spatial models become increasingly feasible (see [1] for review).

The composition of biological membranes is heterogeneous. This heterogeneity is a regulatory mechanism that controls and regulates signaling pathways via location and movement of their components and is reflected in the frequent observation of their subdiffusive behavior [2, 3].

An example of such a specialized environment is the postsynaptic density (PSD). The PSD is located at the postsynaptic side of neuronal synapses, receiving the biochemical signals originating from the presynaptic terminal during neurotransmission. The PSD is a highly organized structure visible in electron microscopy micrographs as an electron dense area in spine heads, the small synaptic protusions found on dendrites [4]. Techniques such as single-particle tracking showed that molecules, including AMPA receptors, exhibit different macroscopic diffusive behavior when they are moving within the PSD or the extrasynaptic membrane [5–7]. The environment of the PSD acts as a trap for receptor molecules depending on synaptic activity, hence regulating synaptic strength [8, 9]. To study these subdiffusive effects in the context of signaling pathways, the membrane environments have to be explicitly represented in the geometry of spatial computer models. This integration should help to better understand the importance of spatially organized and regulated signaling pathways.

Currently, most available simulators [10, 11] require the introduction of an artificial boundary surface surrounding the desired surface area. In recent versions of Smoldyn, a molecule changes its type upon crossing this boundary. This allows a new type of behavior to be specified. However, this comes at the costs of defining the interactions with the boundary surface. This requires the definition of transition rates for molecules crossing the surface. In addition, reactions that take place on all types of surfaces have to be defined for all variants of molecules for each surface type. This results in an explosion of molecule and reaction definitions in the model. This aspect becomes more important as numbers of molecule and surface types increase.

We introduce an extension to the simulation environment Smoldyn that allows to model surface-dependent diffusion. This modified version allows the macroscopic modeling of different diffusive areas of biological membranes. Artificially introduced boundary surfaces intersecting the diffusive surface as described above are not necessary. The modified version Smoldyn is described in the following sections and validated against the original. Examples based on phosphoinositide phosphorylation and AMPAR diffusion in the dendritic spine illustrate its use.

### Implementation

The main modifications to the original Smoldyn source code affect the structures that hold the surface and reaction data. Each surface now holds the information for its specific diffusion coefficients accessible by molecule id and state. Based on this, the functions that calculate reaction parameters such as binding and unbinding radii and diffusion steps can be calculated in a surface dependent manner using the original methods provided by Smoldyn. These modifications do not cause any changes in the control flow of the simulator.

Surface specific diffusion coefficients are defined in the configuration file. A new statement was added to the configuration file syntax to define different surface-dependent diffusion coefficients. This new statement has the form: 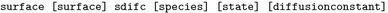 It is meant to be used in addition to the standard Smoldyn difc statement and can be interpreted as adding exemptions to the general diffusive behavior of a molecule species for a specific surface.

In the following, the modified version of Smoldyn will be denoted SmoldynM to distinguish it from the original.

The modified files (smolmolec.c, smolreact.c, smolsurface.c) as well as the complete source code are available online and can be downloaded from GitHub.

### Diffusion

**Fig. 1:**
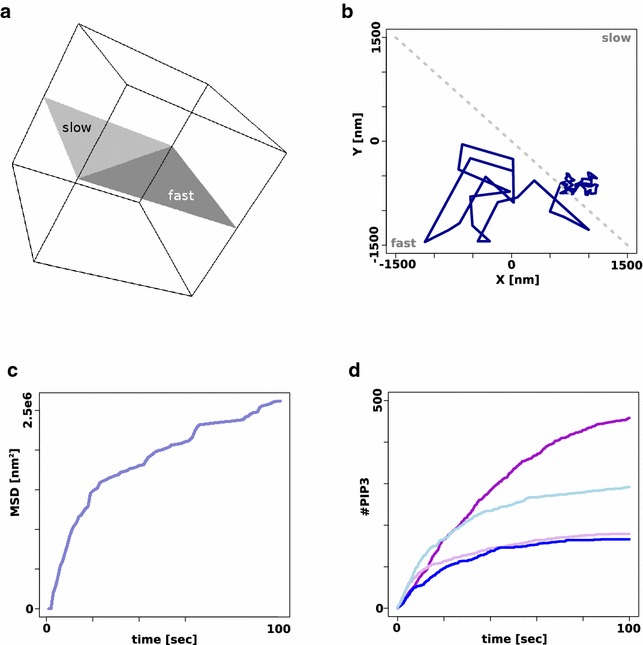
Molecular random walks with surface dependent diffusion. **a**) Geometry of the testing environment. The plane the molecules are diffusing on is separated into two parts. A molecules diffusion coefficient can be set depending on the surface it is diffusing on. **b** Example of a single molecule random walk crossing from a fast diffusion environment to a slow diffusion environment. The *blue line* indicates the path of the molecule, the* grey line* indicates the separation between the fast and the slow diffusing environment. **c** The mean square displacement (MSD) of 20 particles diffusing in different diffusion environments shows anomalous diffusion. **d** Simulations of a simple reaction system using the geometry depicted in **a**). *Purple* Simulations performed using Smoldyn and SmoldynM produce identical results when the simulation setup is identical (same random number seed and therefore seperate simulations for the “fast” (*dark purple*) and “slow” (*light purple*) triangular surface. *Blue*: It is possible to run simulations with SmoldynM where molecules cross over from one triangle to the other and back adopting a new diffusion constant in the process. The development of PIP3 is shown in *dark blue* for the “fast” and *light blue* for the “slow” triangle surface

First, diffusion was tested to show that the new version is working as expected by comparing both Smoldyn versions in scenarios that both simulators can handle. The testing geometry comprises a rectangular plane inside a 3-dimensional box as shown in Fig. 1a. The plane is separated in two triangular panels, each defined as a surface in its own right. The random seeds were fixed to directly compare the diffusion between both Smoldyn versions. Single molecules were set to diffuse for a set amount of time (100 s). Their paths along the surfaces were compared and are identical between simulations using the same seed.

SmoldynM is capable of simulating a single molecule with different diffusion behavior depending on the surface and specific diffusion coefficient can be assigned to each triangle in SmoldynM. Figure 1b shows an example path of a molecule diffusing on the heterogeneous plane illustrated in Fig. 1a (all model files are provided in Additional file 1). The assigned diffusion coefficient is hundredfold faster on the “fast” triangle compared to the “slow” one. The figure clearly shows the different behavior depending on the surface the molecule is currently on.

SmoldynM is expected to run slightly slower than the original version. For n molecular types and m surfaces, lookup tables increase by $\text{ n } \cdot \text{ m }$. Additional operations are simple indexed lookups and should not increase runtimes too much. Running a diffusion simulation with 10,000 molecules over 300 s simulated time and the two triangular surfaces mentioned above, the real-time runtime increases from 617 s to 638 s when using SmoldynM (run on a standard laptop with an Intel i7 CPU and 8GB memory).

The mean square displacement (MSD) [12] of particles was calculated based on 20 simulations tracking one molecule. The MSD can be interpreted as the space explored by particles over time. The MSD of random diffusion is expected to be linear over time. If the molecules are allowed to cross between triangles defining a different diffusion coefficent, the MSD shows the anomalous behavior expected from such a system as shown in Fig. 1c for SmoldynM, indicating subdiffusion, a behavior that is observed for example in systems that exhibit molecular crowding [13].

### Reactions

The same geometry as described before and illustrated in Fig. 1a was used to run a set of simulations to compare reactions in Smoldyn and SmoldynM. The modeled reaction is based on the phosphorylation of the phosphoinositide PIP2 by phosphatidyl-inositol-3-kinase (PI3K). The kinetic parameters where taken from [14]. Diffusion on the “fast” surface is again set up 100x faster than the slow one. Simulations are initiated with 3 kinases and 500 molecules of PIP2 for each triangle. PIP2 is not allowed to move between the fast and slow triangles in the first set of simulation runs and the boundaries of the triangles are reflective.

In this case, direct comparison between Smoldyn and SmoldynM is only possible if it is ensured that the same sequence of random numbers is used during simulation. This required SmoldynM simulations to be run separately for the “fast” and “slow” triangle, mirroring Smoldyn, although simulations using the two different diffusion coefficients for each triangle can run simultaneously using SmoldynM. However, the different number of events by having both triangles populated with molecules and their reactions changes the amount of numbers used for each timestep and would therefore alter the results.

Figure 1d shows the results of these simulations in purple. The development of PIP3 on the “fast” (dark purple) and “slow” (light purple) is identical for Smoldyn and SmoldynM for identical random number seeds. The number of PIP3 is larger on the “fast” surface due to increase chances of PIP2 encountering PI3K.

In addition, simulations were run in SmoldynM connecting the two surfaces, setting the two different diffusion coefficients and allowing PIP2 to cross between surfaces. The same random seed was used as before. The development of PIP3 is shown in blue for both surfaces on Fig. 1d. The trapping of PIP2 molecules on the “slow” diffusion surface causes an increase in the local concentration compared to the “fast” surface. Therefore, more PIP3 is produced on the slow than on the fast surface in this case compared to the other cases where crossing of molecules between surfaces was not possible.

### Example: AMPAR trapping at the PSD

The increase in synaptic AMPAR numbers is one of the major processes of long term potentiation (LTP). Sources for new AMPAR at the synapse are exocytosis and lateral diffusion [15]. This section shows an application for surface dependent diffusion coefficients to study the latter (lateral diffusion) based on a model proposed by [9].

#### Different macroscopic diffusion environments can mimic scaffold binding

Binding partners and mechanisms of detainment of molecules in specific areas of the membrane are often unknown, only vaguely defined or very broad. This can render explicit modeling difficult. Molecular behavior could also be solely influenced by crowded environments. Different macroscopic diffusion environments can be used to abstract from this level of detail as shown for example in Fig. 1a.

**Fig. 2:**
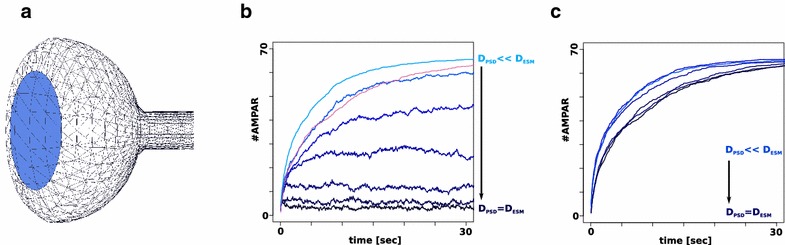
Surface dependent diffusion enables macroscopic simulation of trapping effects at the post synaptic density in dendritic spines. **a** Illustration of the implemented three-dimensional spine geometry. *Light blue* indicates the area of the PSD. **b** Timecourses of AMPAR accumulation at the PSD. The diffusion coefficients (D) for AMPARs at the PSD are changed between timecourses while the diffusion coefficients on the extra synaptic membrane (ESM) are same. D is changed from D$_{\text{ PSD }}$ = D$_{\text{ ESM }}$ (*darkblue*) to D$_{\text{ PSD }}$ $\cdot$ 100,000 = D$_{\text{ PSD }}$ (*lightblue*). All time courses are averages of 10 simulation runs. AMPAR trapping happens solely based on the diffusive properties of the environment. The *pink* time course indicates a different simulation where AMPAR are trapped due to scaffold binding instead of changes in diffusive behavior (D$_{\text{ PSD }}$ = D$_{\text{ ESM }}$). **c** Influence of changes in the diffusive properties of the PSD on scaffold trapping

Based on the model for AMPAR trapping at the PSD in [9] that models scaffold binding of AMPAR explicitly, simulations were run in SmoldynM using different diffusive environments for AMPAR at the PSD and the extra synaptic membrane (ESM). The parameters for the model were taken from [9] (D$_{\text{ ESM }}$ = 0.45 µm$^2$ s$^{-1}$, n(AMPAR) = 66, n(Scaffold) = 132). A more realisitic three-dimensional surface representation of the spine is used as illustrated in Fig. 2a. The enrichment of AMPAR at the PSD is shown for a range of PSD diffusion coefficients of D$_{\text{ PSD }}$ = 0.45 µm$^2$ s$^{-1}$ to D$_{\text{ PSD }}$ = 0.45 10$^{-6}$ µm$^2$ s$^{-1}$ in Fig. 2b. Lighter colors indicate slower diffusion at the PSD. The pink time course indicates AMPAR trafficking due to explicit scaffold binding when D$_{\text{ ESM }}$ = D$_{\text{ PSD }}$ = 0.45 µm$^2$ s$^{-1}$. The figure indicates that the time course of actual scaffold binding resembles trapping due to slower diffusion with D$_{\text{ PSD }}$ = 0.45 10$^{-5}$ µm$^2$ s$^{-1}$. The effects on scaffold binding are explored in more detail in the following section.

#### Different diffusive environments are able to simulate receptor trapping at the synapse

Tolle et al. used explicit modeling of the PSD boundaries to show the effects of AMPAR receptor confinement to the PSD on receptor trapping [9]. They vary the probability that AMPAR are reflected back into the PSD upon encounter of this boundary instead of diffusing into the extra synaptic space. Increased confinement of AMPAR to the PSD decreases the time needed to bind most receptors to the scaffold due to increased probabilities of AMPAR encountering binding scaffold elements. As shown before, a different macroscopic diffusion environment resembles this behavior and enriches receptors at the PSD without the need of explicitly modeling a reflective boundary around it.

The effects of the diffusion environment on explicitly modeled scaffold binding reactions were examined by running SmoldynM simulations with scaffolds while modeling two different diffusive environments for AMPAR. Results are shown in Fig. 2c. The parameters for the model are taken from [9] (*D*$_{\text{ ESM }}$ = 0.45 µm$^2$ s$^{-1}$, n(AMPAR): 66, n(Scaffold) = 132). The extra synaptic diffusion coefficient is the same for all simulations (D$_{\text{ ESM }}$ = 0.45 µm$^2$ s$^{-1}$). The diffusion coefficient for the PSD is changed tenfold from D$_{\text{ PSD }}$ = 0.45 µm$^2$ s$^{-1}$ to D$_{\text{ PSD }}$ = 0.45 10$^{-5}$ µm$^2$ s$^{-1}$. Scaffolding elements and AMPAR binding to them is modeled explicitly. The results indicate the positive effect of slower AMPAR diffusion at the PSD on the overall time that is needed to trap AMPAR by scaffold binding at the PSD. This improvement however has its limitations once diffusion at the PSD gets too slow to enable AMPAR to encounter the scaffold elements.

## Conclusion

We described, validated and illustrated surface-dependent diffusion coefficients as an extension to the simulator Smoldyn.

Examples demonstrate that SmoldynM behaves as expected compared to Smoldyn when such a comparison is directly possible. The new extension enables simulations showing interesting aspects such as anomalous diffusion due to different diffusive areas or the enhancement of reaction speed by enriching substrate molecules.

Simulations of AMPAR trafficking at the PSD show that a change in diffusion coefficient can substitute for explicit modeling of binding reactions, especially in cases where binding partners are unknown, not well defined or an abundance of unspecific associations slows the movement of molecules down. Especially in case of the latter, modeling the behavior based on macroscopic diffusive behavior might be the easier and also biologically more realistic abstraction. It has to be kept in mind that the results are not the same with regards to the spatial distribution of the trapped molecules. Modeling molecular trapping by unspecified scaffolds defines the position of receptors via the positioning of the scaffold. Trapping them via a change in diffusivity initially concentrates them on the boundaries of the subarea. Achieving a random or uniform distribution of molecules via macroscopic diffusion in that subarea might take longer since the molecules have to distribute within this subarea via the slower diffusion coefficient.

In general, being able to easily model membranes as surfaces composed of subareas that exhibit different properties is an important feature to study the influence of special subdiffusive membrane environments like the PSD.

## Availability and requirements

- Project name: SmoldynM
- Project source code The extended Smoldyn can be found at http://lenoverelab.org/documents/smoldynM-2.22s.tar.gz
- Operating System: Platform independent
- Programming Language: C
- Other requirements: None
- License: GPL
- Any restrictions to use by non-academics: No

## Additional file

10.1186/s13104-015-1723-6 Smoldyn configuration files used in this manuscript.
